# Highlighting Indication of extracorporeal membrane oxygenation in endocrine emergencies

**DOI:** 10.1038/srep13361

**Published:** 2015-08-24

**Authors:** Anne Chao, Chih-Hsien Wang, Hao-Chun You, Nai-Kwoun Chou, Hsi-Yu Yu, Nai-Hsin Chi, Shu-Chien Huang, I-Hui Wu, Li-Jung Tseng, Ming-Hsien Lin, Yih-Sharng Chen

**Affiliations:** 1 Department of Anesthesiology, National Taiwan University Hospital, College of Medicine, National Taiwan University Hospital, Taipei, Taiwan; 2 Department of Surgery, National Taiwan University Hospital, College of Medicine, National Taiwan University Hospital, Taipei, Taiwan

## Abstract

Extracorporeal membrane oxygenation (ECMO) has been repeatedly used to rescue patients with cardiopulmonary arrest. However, its clinical utility in endocrine emergencies remains unclear. Herein, we describe a case series of 12 patients presenting with refractory shock secondary to endocrine emergencies who were rescued by ECMO support. Patients were identified between 2005 and 2012 from our ECMO registry. The diagnostic distribution was as follows: pheochromocytoma crisis (n = 4), thyroid storm (n = 5), and diabetic ketoacidosis (n = 3). The initial presentation of pheochromocytoma crisis was indistinguishable from acute myocardial infarction (AMI) and frequently accompanied by paroxysmal hypertension and limb ischemia. Thyroid storm was characterized by hyperbilirubinemia and severe gastrointestinal bleeding, whereas neurological symptoms were common in diabetic ketoacidosis. The clinical outcomes of patients with endocrine emergencies were compared with those of 80 cases with AMI who received ECMO because of cardiogenic shock. The cardiac function and the general conditions showed a significantly faster recovery in patients with endocrine emergencies than in those with AMI. We conclude that ECMO support can be clinically useful in endocrine emergencies. The screening of endocrine diseases should be considered during the resuscitation of patients with refractory circulatory shock.

Extracorporeal membrane oxygenation (ECMO) is a treatment used to temporarily replace the function of the heart and/or lungs over an extended period of time to allow for organ recovery. Several studies have reported the successful use of ECMO to rescue patients with cardiopulmonary arrest in a wide spectrum of different etiologies, including acute respiratory distress syndrome^1^, drug intoxication^2^, acute myocarditis^3^, burns^4^, acute myocardial infarction (AMI)^5^, post-cardiotomy shock^6^, and severe cardiomyopathy^7^,^8^.

Endocrine emergencies – including pheochromocytoma crisis (PC), thyroid storm (TS), and diabetic ketoacidosis (DK) – are rare but potentially life-threatening conditions if not recognized early and managed properly. The treatment of endocrine emergencies remains challenging even with the armamentarium of modern intensive care technologies, especially in patients with cardiopulmonary failure and major organ dysfunction. Although ECMO may provide mechanical pulmonary and circulatory support in patients refractory to conventional therapies, to date only a few cases of its use in endocrine emergencies have been reported in the literature^9^,^10^,^11^,^12^,^13^,^14^,^15^,^16^. Herein, we describe a case series of 12 patients presenting with refractory shock secondary to endocrine emergencies who were successfully rescued by ECMO support. We also compared their clinical outcomes with those of 80 cases with AMI who received ECMO because of cardiogenic shock.

## Methods

Study approval was obtained by our Institutional Review Board (No. 201404079 RIN), which waived the requirement for informed consent because of the retrospective nature of the study. Among the patients aged 16 years or older who received ECMO and reported to our hospital-based ECMO registry during the period January 2005–December 2012, we retrospectively reviewed the data of those who presented with refractory shock due to endocrine emergencies. The indications for ECMO included catecholamine-refractory shock and failed conventional cardiopulmonary resuscitation. The circuit and management of ECMO have been described previously^5^,^8^. Following completion of ECMO, presumed diagnoses were confirmed by reviewing all of the clinical and laboratory data. Because some cases were initially incorrectly diagnosed, a careful analysis of all clinical records was performed before including the patient in the study.

We identified 12 cases presenting with refractory shock secondary to endocrine emergencies successfully rescued by ECMO support. The diagnostic distribution was as follows: PC (n = 4), TS (n = 5), and DK (n = 3). PC was identified through plasma and urinary catecholamines and metanephrines testing and imaging confirmation of adrenal lesions compatible with pheochromocytoma. TS was diagnosed on the basis of serum thyroid hormone levels and traditional signs and symptoms of a thyrotoxic state. The diagnosis of DK was made in presence of uncontrolled hyperglycemia, metabolic acidosis, and increased total blood ketone concentrations, either with or without a history of type 1 diabetes mellitus. The following variables were collected in all participants: age, sex, initial clinical presentation, final diagnosis, time taken to identify the etiology, initial sepsis-related organ failure assessment (SOFA) score^17^, ECMO duration, pre- and post-ECMO inotropic equivalent (IE = dopamine × 1 + dobutamine × 1 + norepinephrine × 100 + epinephrine × 100, all expressed in μg/kg/min), post-ECMO blood pressure values, biochemical data, hormone levels, and length of stay in the intensive care unit. The main outcome measures included neurological conditions at discharge, survival, duration of ECMO, and the occurrence of complications after ECMO. Because the clinical presentation of several patients with endocrine emergencies closely resembled AMI, we compared their general characteristics and outcomes with those of 80 cases with AMI (aged ≤65 years) who received ECMO because of cardiogenic shock.

## Results

**Figure 1:**
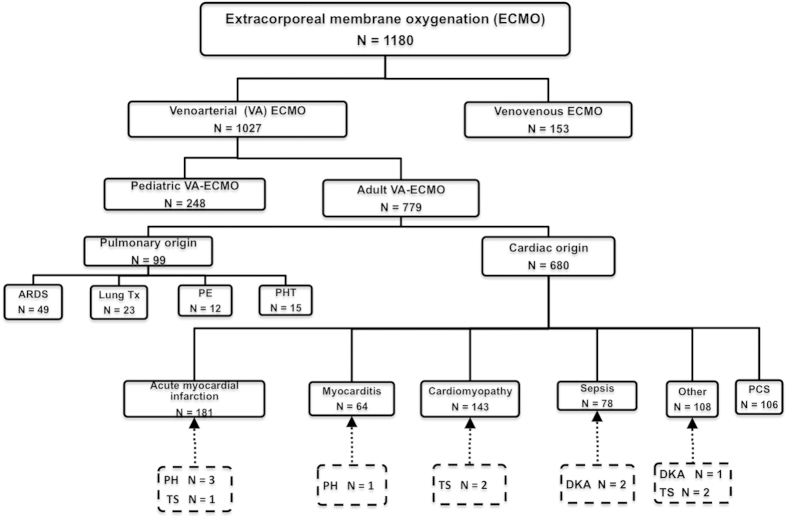
Flow diagram of patients with endocrine emergencies who were rescued by extracorporeal membrane oxygenation. Abbreviations: ARDS: acute respiratory distress syndrome; AMI: acute myocardial infarction; CMP: cardiomyopathy; DK: diabetic ketoacidosis; ECMO: extracorporeal membrane oxygenation; PCS: post-cardiotomy shock; PE: pulmonary embolism; PC: pheochromocytoma crisis; PHT: pulmonary hypertension; TS: thyroid storm; Tx: lung transplantation; VA: veno-arterial; VV: veno-venous.

The flow of patients through the study is depicted in Fig. 1. During the period January 2005–December 2012, a total of 1180 patients were rescued with ECMO [1027 with veno-arterial (VA) ECMO and 153 with veno-venous (VV) ECMO] according to our registry data. We identified 12 patients presenting with refractory shock secondary to endocrine emergencies (PC, n = 4; TS, n = 5; and DK, n = 3) who were rescued by ECMO support. The clinical outcomes of patients with endocrine emergencies were compared with those of 80 cases with AMI who received ECMO because of cardiogenic shock. Endocrine emergencies represented 1% of all cases treated with ECMO (1.2% of VA ECMO). The clinical course is presented separately for each endocrine emergency.

### Pheochromocytoma crisis

We identified four patients with PC. The presentation of PC mimicked AMI with palpitations, chest pain, abnormal electrocardiographic findings, and elevations of cardiac enzymes (Tables 1 and 2). Not surprisingly, most patients with PC were initially misdiagnosed as having an acute coronary syndrome. Three PC patients (PC-1, PC-2, and PC-4) had a bystander-witnessed out-of-hospital cardiac arrest and ECMO was initiated during cardiac pulmonary resuscitation (CPR) in the emergency department. Patient PC-3 presented with an intractable cardiogenic shock requiring ECMO to allow for organ recovery. Paroxysmal hypertension was identified in all of these patients following ECMO implantation. All of the patients in the PC group underwent coronary angiography. Patients PC-1, PC-2, and PC-3 had normal coronary angiography, which led to the suspicion of pheochromocytoma. Because of a 50% stenosis in the left circumflex artery, patient PC-4 was initially treated as having an AMI and weaned off ECMO support after 48 h under stable hemodynamic conditions. An episode of pulseless ventricular tachycardia occurred 2 h later, requiring resumption of CPR and ECMO. Because of the extreme blood pressure fluctuations, a diagnosis of PC was suspected. Patients PC-1 and PC-3 experienced a severe lower leg compartment syndrome despite placement of a distal perfusion catheter to prevent limb ischemia. In patient PC-1, the femoral-femoral VA ECMO was converted to a central ECMO after median sternotomy avoiding further worsening of limb ischemia. Patient PC-3 underwent a below-knee amputation of the left leg. The patients received abdominal computed tomography scans to localize the tumor and confirm the diagnosis. All PC patients underwent adrenalectomy after discharge from the intensive care unit, the only exception being PC-4 who refused surgery.

### Thyroid storm

Five patients with TS were identified throughout the study period. All of them had a history of hyperthyroidism, the only exception being TS-1 (in whom TS was suspected because of normal angiographic findings). Most patients with TS were initially misdiagnosed as having AMI or severe congestive heart failure. Their rapid clinical deterioration required emergency ECMO support. Hyperbilirubinemia was evident in all of the TS patients, whereas patient TS-2 and TS-4 developed massive gastrointestinal bleeding. Under ECMO support and anti-thyroid drug therapy, the cardiac function of four of the 5 TS patients started recovering between day 3 and day 4. They were subsequently weaned off ECMO. Patient TS-1 died of multi-organ failure while receiving ECMO, whereas patient TS-2 eventually died of liver failure. Patient TS-4 suffered from Graves’ ophthalmopathy resulting in exposure keratopathy of the left eye, which eventually required penetrating keratoplasty.

### Diabetic ketoacidosis

All of the three patients with DK were found unresponsive at home. Patients DK-1 and DK-3 had a diagnosis of type 1 diabetes mellitus, but their compliance with insulin therapy was poor. Patient DK-2’s relatives denied any known medical history. Patients DK-1 and DK-2 were admitted to the emergency department with out-of-hospital cardiac arrest. Patient DK-3 required ECMO because of profound hypothermia and hypotension unresponsive to aggressive fluid replacement and high-dose catecholamine administration. The presence of uncontrolled hyperglycemia, metabolic acidosis, and increased total blood ketone concentrations led to a diagnosis of DK. Because patient DK-1 was also diagnosed with community-acquired pneumonia that progressed to acute respiratory distress syndrome, both VA and VV ECMO support were indicated. VA and VV ECMO were stopped 200 h and 381 h after initiation, respectively. The patient eventually developed gangrene of the distal parts of all four limbs and died of septic shock and multiple organ failure. Spontaneous circulation in patient DK-2 did not return despite ECMO support. Patient DK-3 was successfully resuscitated and eventually recovered.

### Comparison between endocrine emergencies and AMI

PC and TS were frequently undistinguishable from AMI at presentation. Despite lower sepsis-related organ failure assessment (SOFA) scores in the AMI group, heart function and clinical outcomes did not differ significantly from those of patients with endocrine emergencies. AMI patients required higher doses of inotropic agents to stabilize their hemodynamic status and their left ventricular ejection function remained poor. The ECMO weaning success rates for patients with AMI and endocrine emergencies were 70% and 83%, respectively. Patients with AMI required the following subsequent interventions: coronary bypass surgery (n = 26), repair of a ruptured ventricular septal defect (n = 4), implantation of a left ventricular assist device (n = 3), cardiorrhaphy following removal of ECMO, and heart transplantation (n = 1). The neurological outcomes did not differ significantly between the two groups. Eighty percent of the patients who survived an AMI had a cerebral performance category (CPC) score of 1, which was found in 83% of those who survived an endocrine emergency (Table 3).

## Discussion

To the best of our knowledge, this report constitutes the largest series to date describing the outcomes of patients presenting with refractory shock caused by endocrine emergencies (i.e., PC, TS, and DK) rescued by ECMO support. A total of 12 cases were identified in our registry from 2005 to 2012, indicating that the incidence of endocrine emergencies is low but not negligible. Pheochromocytoma has been estimated to be present in approximately 0.3% of all patients undergoing evaluation for secondary causes of hypertension^18^. Moreover, TS and DK accounts for 2% and 8.6% of all hospital admissions in patients with thyrotoxicosis and type 1 diabetes mellitus, respectively^19^,^20^. In general, endocrine emergencies can have a rapid and aggressive clinical course and pose significant diagnostic challenges (because they can mimic either AMI or acute congestive heart failure). Endocrine emergencies are not common and probably misdiagnosed in many cases, and the clinical value of ECMO support in their clinical management remains unclear. Notably, following our report of a patient with PC who was successfully rescued by ECMO in January 2008^9^, seven additional cases were described (a finding that illustrates the magnitude of the potential underestimation as well as a recent increase in awareness)^10^,^11^,^12^,^13^,^14^,^15^,^16^.

In the current series, all patients with PC did not have a known history of pheochromocytoma. The main diagnostic features of PC consisted of extreme blood pressure fluctuations in presence of patent coronary arteries on angiography. Compared with AMI patients, inotropic agents were more rapidly tapered and myocardial function recovered significantly more quickly in PC. However, two of the four patients with PC (50%) developed limb ischemia, a percentage which was significantly higher than those observed in both the DK (one patient, 33%) and AMI (six patients, 7.5%) groups (Table 3). To our knowledge, a total of 12 cases of pheochromocytoma-associated peripheral limb ischemia have been reported to date^21^. In this scenario, early recognition and treatment of pheochromocytoma through adrenalectomy or alpha-adrenergic blocking agents is paramount to reduce the risk of devastating limb ischemia.

In this series, most patients with TS were initially misdiagnosed as having AMI or severe congestive heart failure. Hyperbilirubinemia was a common finding in TS patients, most likely caused by a direct hepatotoxic effect of excess thyroid hormones and/or hepatic congestion resulting from thyroid storm. Although anti-thyroid drugs (e.g., propylthiouracil) can cause cholestatic liver injury, previous studies conducted in TS patients have shown that jaundice can successfully respond to anti-thyroid medications^22^,^23^. Consequently, the timely detection of TS is crucial to the successful preservation of liver function.

Blood glucose levels are routinely checked in all of the patients admitted to our emergency department with disturbances of consciousness. The diagnosis of DK can be easily established upon admission in presence of hyperglycemia, metabolic acidosis, and increased serum or urine ketones. In general, delays to seek medical care and the presence of sepsis and/or coma have adverse consequences for DK patients’ clinical outcomes.

In the current study, AMI patients had lower SOFA scores and higher levels of cardiac enzymes than those with endocrine emergencies. Notably, the left ventricular ejection function and the clinical outcomes were poorer in patients with AMI than in those with endocrine emergencies rescued by ECMO (Table 3). Similarly, the number of patients who required long-term inotropic agents was higher in the AMI group.

Taken together, there are three main findings from the current single-center study: 1) endocrine emergencies are rare but not negligible events, 2) patients with endocrine emergencies presenting with acute refractory shock can be successfully rescued by ECMO support, and 3) screening of endocrine emergencies is essential in patients with acute refractory shock requiring mechanical support. Albeit preliminary in nature because of the small sample size, our results may stimulate further studies on the cost-benefits analysis of routine screening of thyroid hormones, catecholamines, and metanephrines in patients presenting with acute circulatory collapse in need of ECMO support.

## Conclusions

Successful management of endocrine emergencies depends on prompt recognition, correction of the underlying hormone alterations, and immediate treatment of shock. Unfortunately, numerous patients presenting with endocrine emergencies have an unknown previous history of hormone imbalance. Therefore, the screening of endocrine emergencies is important in these patients. Herein, we have shown that ECMO support is clinically useful in patients diagnosed with endocrine emergencies presenting with acute circulatory failure. Because medical care is extremely challenging in this scenario, the awareness of the clinical value of ECMO among physicians managing patients with endocrine emergencies may help improve clinical outcomes.

## Additional Information

**How to cite this article**: Chao, A. *et al.* Highlighting indication of extracorporeal membrane oxygenation in endocrine emergencies. *Sci. Rep.* **5**, 13361; doi: 10.1038/srep13361 (2015).

## Figures and Tables

**Table 1:** Demographic and clinical characteristics of patients with endocrine emergencies who were rescued by extracorporeal membrane oxygenation.

**Case**	**Age (y)**	**Sex**	**Initial presentation**	**Past history**	**Initial diagnosis**	**Day of diagnosis**	**Endocrine data**	**Survival**
Pheochromocytoma crisis							Urine VMA/Dopa/Epi/NE	
PC1	25	F	Dyspnea, palpitations, cold sweats	Myocarditis	AMI/myocarditis	28	15.3/18/51/28.3	Yes
PC2	52	M	Chest pain	Hypertension	AMI	6	20.7/217/146/858	Yes
PC3	40	M	Chest pain	Hypertension	AMI	3	64.3/498.3/3.34/216.2	Yes
PC4	65	M	Chest pain, palpitations	HBV carrier	AMI	4	11/278.7/9/120.8	Yes
Diabetic ketoacidosis							Blood glucose/ketone bodies	
DK1	16	F	Fever, loss of consciousness	T1DM	Septic shock	1	770/+	No
DK2	34	M	Fever, drowsiness	Nil	Septic shock	1	1505/5.9	No
DK3	28	F	Loss of consciousness	T1DM	DK	1	994/3.6	Yes
Thyroid storm							Free T4/T3/TSH	
TS1	47	M	Palpitations, exertional dyspnea	Gout	AMI	3	4.26/92.7/0.04	No
TS2	43	M	Dyspnea	Graves’ disease, alcohol liver	Thyroid storm/CHF	4	2.42/237/0.03	No
TS3	37	F	Dry cough, night sweating, alteration of consciousness	Graves’ disease	CHF	1	7.5/-/0.03	Yes
TS4	42	M	Palpitations, dyspnea, nausea, delirium	Graves’ ophthalmopathy	Thyroid storm	1	24/7.22/<0.01	Yes
TS5	33	F	Fever, shortness of breath	Hyperthyroidism	Thyroid storm	1	4.5/425/<0.01	Yes

Abbreviations: M: male; F: female; y: years; HBV: hepatitis B virus; AMI: acute myocardial infarction; T1DM: type 1 diabetes mellitus; CHF: congestive heart failure; Dopa: dopamine (normal range: 50–450 μg·day^−1^); Epi: epinephrine (normal range <22.4 μg·day^−1^); NE: norepinephrine (normal range 12.1–85.5 μg·day^−1^); free T4: thyroxine (normal range: 0.89–1.76 ng/dL); T3: triiodothyronine (normal range 84–172 ng/dL); TSH: thyroid-stimulating hormone (normal range 0.4–4 μIU/mL); VMA: vanillomandelic acid (normal range 1–7 mg·day^−1^).

**Table 2:** Clinical characteristics of patients with endocrine emergencies at the time of ECMO implantation.

	**PC (n = 4)**	**DK (n = 3)**	**TS (n = 5)**
SOFA score (range)	13. (9–18)	13.7 (8–17)	15 (13–18)
Extracorporeal cardiopulmonary resuscitation, n	3	2	3
Inotropic index, μg/kg/min	28.3	56.8	35.5
IABP, n	2	1	2
pH (range)	7.30 (7.1–7.36)	7.12 (6.63–7.39)	7.24 (7.14–7.39)
Blood lactate, mmol/L (range)	10.3 (8.7–11)	6.25 (5.0–7.5)	9.6 (3.5–12)
Initial CK, U/L (range)	1572.5 (501–3796)	225.3 (348–2576)	714.3 (259–1574)
Initial CK-MB, U/L (range)	86.2 (15–290)	21.1 (14–32)	42.6 (21–75)
Initial troponin-I, μg/L (range)	37.0 (0.9–79)	0.58 (0.5–0.7)	0.35 (0.02–1.1)
Serum sodium, mEq/L (range)	141.3 (138–144)	146.3 (131–155)	143 (128–149)
Serum BUN, mg/dL (range)	16.9 (14–24.9)	47.1 (37.5–52.4)	29.4 (19.6–75.5)
Serum creatinine, mg/dL (range)	2.1 (0.8–2.8)	2.3 (2.1–2.6)	1.6 (1.1–2.40)
Serum bilirubin, mg/dL (range)	0.9 (0.4–1.40)	0.67 (0.12–1.5)	7.2 (2.6–14.50)
Serum AST, U/L (range)	643.5 (52–2183)	109 (15–239)	2464.5 (195–6195)
Serum ALT, U/L (range)	657.2 (47–2183)	43 (10–73)	761 (44–2267)

Abbreviations: AST: aspartate aminotransferase; ALT: alanine aminotransferase; BUN: blood urea nitrogen; CK: creatine kinase; DK: diabetic ketoacidosis; IABP: intra-aortic balloon pump; PC: pheochromocytoma crisis; SOFA: sepsis-related organ failure assessment; TS: thyroid storm.

**Table 3:** Comparisons of patients with endocrine crisis and acute myocardial infarction rescued with ECMO support.

	**PC (n = 4)**	**DK (n = 3)**	**TS (n = 5)**	**AMI (n = 80)**
Age, years (range)	45.5 (25–65)	26 (16–34)	40.4 (33–47)	53.8 (31–65)
Male sex, n (%)	3 (75)	1 (33.3)	3 (60)	75 (94)
CPR, n (%)	3 (75)	2 (66)	3 (60)	55 (68.7)
ECMO duration, hour (range)	102.5 (44.7–162.5)	134.5 (0.9–381.8)	82 (19–115.6)	117 (5.4–475)
Ventilator support, day (range)	14 (6–22)	17.7 (1–51)	10 (4–26)	17.7 (1–95)
ICU length of stay, day (range)	20.8 (6–28)	18.0 (0–51)	11.8 (5–26)	21.6 (1–147)
Initial SOFA score (range)	13 (9–18)	13.7 (8–17)	15 (13–18)	10.4 (3–18)
MAP Day 1, mmHg (range)	120 (101–137)	97 (96–99)	84 (63–101)	74 (30–144)
Day 3 (range)	121 (91–146)	85 (84–86)	113 (104–126)	76 (920–112)
Day 6 (range)	130 (116–145)	99 (99)	114 (93–132)	88 (61–118)
Pre-ECMO IE, μg/kg/min	28.3	56.8	35.5	21.4
IE, day 3 (range)	4.7 (2.1–7.0)	32.4 (32.4)	4.6 (0–18.4)	24.1 (0–810)
IE, day 6 (range)	1.4 (0–5.4)	11.5 (11.5)	0 (0)	8.2 (0–31.8)
Patients using inotropes at day 6, n, (%)	1 (25)	1 (33%)	0 (0%)	50 (62.5%)
Initial CK, U/L (range)	1572.5 (501–3796)	225.3 (348–2576)	714 (259–1574)	2413 (41–28480)
CK day 3 (range)	14589 (1528–47829)	38.5 (38.5)	8364.5 (515–4446)	5808.4 (77–66274)
CK day 6 (range)	22637 (530–13721)	4496 (4496)	501 (114–888)	3163.4 (33–36621)
Initial CK-MB, U/L (range)	86.2 (15–290)	21.1 (14–32)	42.6 (21–75)	172 (4.2–2319.8)
CK-MB day 3 (range)	3892 (90.9–10031)	52.2 (52.2)	53.5 (39.6–64.6)	132.1 (1.5–1782)
CK-MB day 6 (range)	66 (15.7–207)	26 (26)	45.1 (40.7–49.4)	50.4 (1.2–308.9)
Initial Tn-I, *μ*g/L (range)	37.0 (.9–79)	0.58 (0.5–0.7)	0.35 (0.02–1.1)	19 (0.01–100)
Tn-I day 3 (range)	16.1 (9.4–24.4)	0.4 (0.4)	1.2 (0.5–1.8)	37.7 (0.44–94)
Tn-I day 6 (range)	2.2 (1.26–3.1)	Not available	Not available	16.1 (0.31–33)
Lactate in 24 h, mmol/L (range)	10.3 (8.7–11)	6.25 (5.0–7.5)	9.6 (3.5–12)	8.9 (1–35)
Dialysis patients, n	3	1	1	36
IABP (at implantation)	2	1	1	25 (32%)
Bilirubin day 3, mg/dL (range)	1.3 (1.0–1.9)	0.9 (0.9)	10.6 (2.0–24.8)	2.5 (0.4–12.2)
Bilirubin day 6 (range)	1.7 (1.4–1.8)	5.5 (5.5)	19.5 (5.67–26.9)	3.5 (0.6–21.3)
LVEF day 2, % (range)	37.7 (30–43)	54 (54)	24 (20–40)	33 (7–68)
LVEF day 6, % (range)	61 (50–67)	62 (62)	55 (38–64)	37.4 (18–65)
Weaned off ECMO, (%)	4 (100%)	2 (66.7%)	4 (80%)	56 (70%)
Complications				
Limb ischemia	2	1	0	6
Massive GI bleeding	0	1	2	1
Corneal erosion	0	0	1	0
ARDS/Pneumonia	0	1	0	8
Reinstitution of ECMO	1	0	0	1
Severe brain insult				23
Further intervention				
CABG, n	0	0	0	26
Repair VSD/wall, n	0	0	0	4
VAD, n	0	0	0	3
Heart transplantation, n	0	0	0	1
Adrenalectomy, n	3	0	0	0
Cause of mortality				
Sepsis-related MOF	0	1	0	7
Hepatic failure, MOF	0	0	1	0
Cardiogenic shock	0	1	1	28
Brain death	0	0	0	3
ARDS	0	0	0	2
Survival to discharge (%)	4 (100%)	1 (33.3%)	3 (60%)	40 (50%)
Survivors with CPC I, n (%)	4 (100% )	1 (100%)	2 (66.7%)	32 (80%)

Abbreviations: AMI: acute myocardial infarction; ARDS: acute respiratory distress syndrome; CABG: coronary artery bypass surgery; CK: creatine kinase; CPC: cerebral performance category; CPR: cardiac pulmonary resuscitation; DK: diabetic ketoacidosis; GI: gastrointestinal; IABP: intra-aortic balloon pumping; ICU: intensive care unit; IE: inotropic equivalents; LVEF: left ventricular ejection fraction; MAP, mean arterial pressure; MOF: multiple organ failure; PC: pheochromocytoma crisis; SOFA: sepsis-related organ failure assessment; Tn-I: troponin-I, TS: thyroid storm; VAD: ventricular assist device; VSD: ventricular septal defect.
